# Acute Hypoxic Respiratory Failure and Septic Shock Secondary to Mycoplasma Pneumoniae Pneumonia Complicated by Heroin Overdose

**DOI:** 10.7759/cureus.11093

**Published:** 2020-10-22

**Authors:** Jesse C Wu, Matthew Solomon, Amanda L Webb McAdams, Latha Ganti

**Affiliations:** 1 Emergency Medicine, University of Central Florida College of Medicine, Orlando, USA; 2 Emergency Medicine, Osceola Regional Medical Center, Kissimmee, USA; 3 Emergency Medicine, Brown University, Providence, USA; 4 Emergency Medicine, Brandon Regional Hospital, Brandon, USA; 5 Emergency Medicine, Envision Physician Services, Nashville, USA; 6 Emergency Medical Services, Polk County Fire Rescue, Bartow, USA

**Keywords:** intravenous drug use, tobacco, heroin, hypoxic respiratory failure, septic shock, mycoplasma pneumoniae, pneumonia

## Abstract

We present the case of a woman with a past medical history of intravenous drug use and tobacco abuse who was brought into the emergency department by emergency medical services after a heroin overdose. She was found to be in acute hypoxic respiratory failure and developed septic shock secondary to Mycoplasma pneumonia. In this case report, the presentation and management of fulminant Mycoplasma pneumonia are discussed, along with plain chest radiography findings.

## Introduction

*Mycoplasma pneumoniae* is one of the most common causes of upper respiratory infection (URI), acute bronchitis, and community-acquired pneumonia (CAP) [1]. The infection is transmitted from person-to-person through respiratory droplets and has an average incubation time between two and three weeks. Outbreaks typically occur in people living in close quarters, such as schools, homes, and military barracks [2]. *Mycoplasma pneumoniae *lacks a cell wall, and therefore it cannot be visualized by gram stain. Additionally, it is not sensitive to antibiotics, such as penicillin, which targets cell wall synthesis. URI and bronchitis are the most common presentation of *M. pneumoniae* infection. The patient typically presents with cough, rhinorrhea, rhinitis, and pharyngitis [3]. Less commonly, *M. pneumoniae* can present as CAP with mild fever, headache, malaise, cough, shortness of breath, and pleuritic chest pain. The pneumonia is typically mild and self-limited. Patients usually recover completely even without antibiotics. In rare cases, fulminant pneumonia can develop, resulting in respiratory failure and necessitating pulmonary support, targeted antibiotics therapy, and microbiological diagnosis by polymerase chain reaction, nucleic acid amplification tests, or serology [4].

## Case presentation

**Figure 1:**
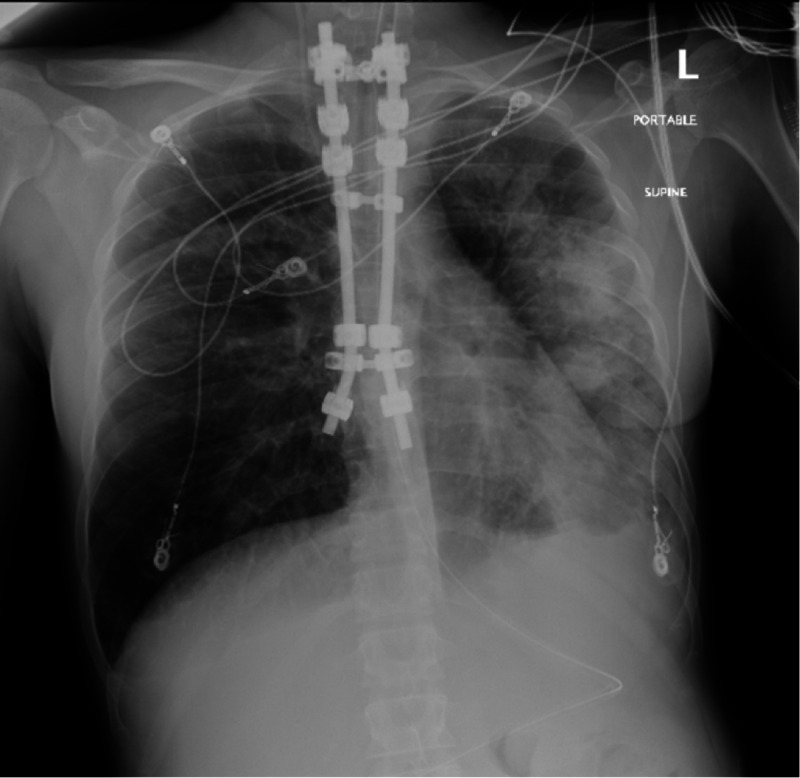
Hospital day 1: multiple patchy opacities in the left lung, representing pneumonia. A small left pleural effusion is also present.

A 34-year-old woman with a past medical history of intravenous (IV) drug use and tobacco abuse was brought to a free-standing emergency department (ED) by the emergency medical services (EMS) for heroin overdose. She was lethargic and was given two doses of naloxone by EMS with some improvement in her level of consciousness. She was given another dose of naloxone in the ED and was put on 2 L per minute oxygen by nasal cannula due to hypoxia. However, she remained somnolent although arousable. Her oxygen saturation improved to 95%. She was tachycardic with a rate of 114 beats per minute (bpm). Otherwise, she was afebrile, and her blood pressure and respiratory rate were within normal limits. Her white blood cell (WBC) count was normal, and her urine drug screen was positive for opiates, amphetamines, and cocaine. She received 2 L of normal saline and ceftriaxone prophylaxis against aspiration pneumonia, and was transferred to the main ED. On arrival, she was found to be febrile (102°F) and tachycardic. She received another bolus of normal saline and one more dose of naloxone with mild improvement in her mental status. The following morning she desaturated and her heart rate increased to 140 bpm. She was placed on a non-rebreather mask with improvement in her oxygen saturation. Chest radiograph performed at the time showed bilateral airspace opacities, suggestive of pneumonia (Figure 1).

**Figure 2:**
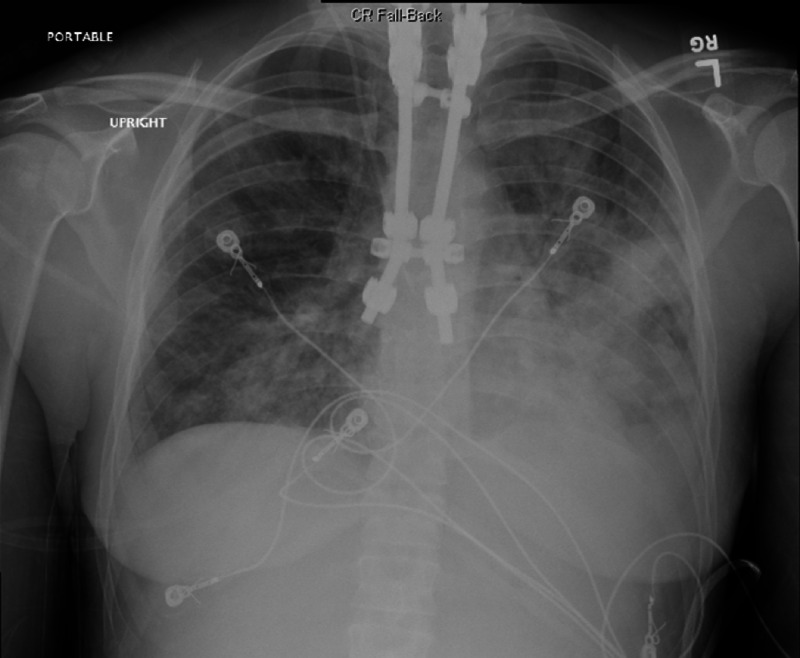
Hospital day 2: worsening of the left lung field airspace opacities and right lower lobe airspace opacity, representing interval worsening of bilateral pneumonia.

However, she desaturated again to 80% and was found to be hypotensive to 88/44 mmHg. She was responsive to pain only. Rapid response was called, and the patient was intubated for airway protection and acute hypoxic respiratory failure. She was then transferred to the intensive care unit (ICU) for further care and management. The patient was mechanically ventilated and was started on vancomycin and piperacillin-tazobactam, IV fluid support, and a norepinephrine infusion. Her lactic acid was 1.8 mmol/L, procalcitonin was 44.04 ng/mL, and WBC count was 13.97 x 10³. She remained stable in the ICU and was gradually weaned off the ventilator and pressor. She was extubated the next morning. On hospital day 2, she still required IV fluid support to maintain mean arterial pressure (MAP) above 65 mmHg, and she was still tachycardic at 112 bpm. Otherwise, her fever resolved and she was saturating normally on 5 LPM oxygen by nasal cannula. She had bilateral rales and rhonchi on examination. Her leukocytosis resolved, but her procalcitonin increased to 120.60 ng/mL. Her repeat chest radiograph showed worsening consolidation bilaterally, and her *M. pneumoniae *IgM was positive (Figure 2).

**Figure 3:**
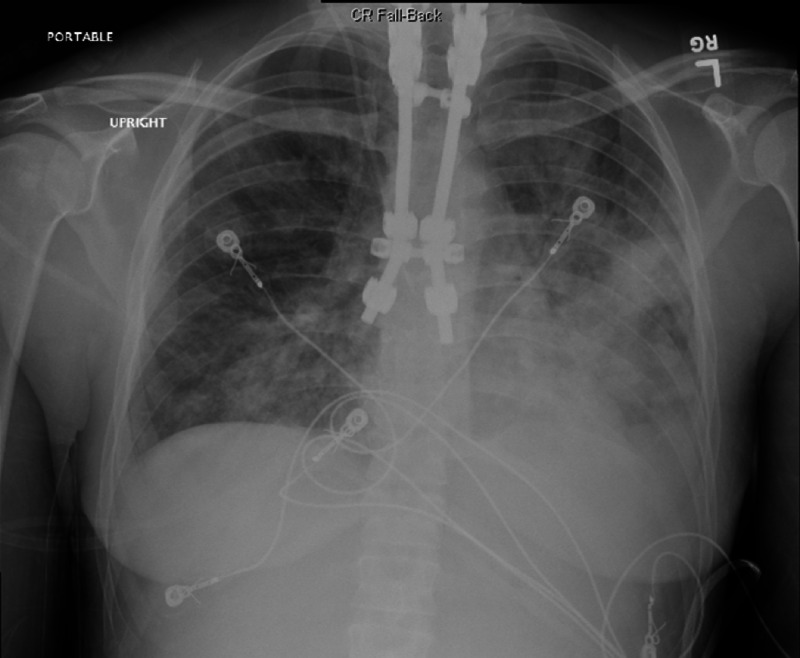
Chest radiograph on hospital day 5 showing interval decrease in bilateral airspace opacities, representing an improvement in bilateral pneumonia.

She was started on azithromycin for presumptive bilateral *M. pneumoniae* pneumonia (MPP). Further workup revealed that she was human immunodeficiency virus (HIV) negative. Urine antigens were negative for *Streptococcus* or *Legionella*. Sputum cultures and urinalysis were additionally negative for any bacteria. However, the patient was found to have chronic hepatitis B (hepatitis B surface antigen and core IgG positive, surface antibody and core IgM negative). Transthoracic echocardiogram revealed no vegetations, and the ejection fraction was 65-75%. On hospital day 3, her blood pressure improved, with an MAP of 80-90 mmHg. She was gradually weaned off nasal cannula, maintained a normal oxygen saturation, and her heart rate normalized throughout the day. Her repeat procalcitonin was 66.32 ng/mL. On hospital day 4, vancomycin and piperacillin-tazobactam were discontinued due to negative workup for other sources of infection. The patient continued to improve on hospital day 5 with interval decreased in bilateral pneumonia on chest X-ray (Figure 3).

She was eventually discharged in stable condition on hospital day 7, after a five-day course of azithromycin.

## Discussion

MPP infection is typically mild; however, in rare cases, fulminant infection with respiratory failure can result, especially in young adults. Fulminant cases are estimated to be 0.5-2% of all MPP cases, with mortality reported to be 3-5% in the 1980s [4]. Clinical features include fever, cough, dyspnea, and bilateral infiltrates and pleural effusion on chest X-ray. Laboratory findings include elevated WBC count, C-reactive protein, lactate dehydrogenase, aspartate transaminase, and alanine transaminase. First-line treatment for MPP includes macrolides (e.g. azithromycin) with tetracyclines (e.g. doxycycline). Second-line agents include respiratory fluoroquinolones (e.g. levofloxacin or moxifloxacin), which are reserved for macrolide-resistant cases [5].

To date, there are several reported cases of fulminant MPP resulting in respiratory failure [6-7]. However, there are no reports of MPP with respiratory failure complicated by opiate overdose. In our case presentation, the patient presented with somnolence, dyspnea, tachycardia, and fever in the setting of heroin overdose. The differential diagnosis can be broad, including alcohol withdrawal, thyroid storm, anticholinergic or serotonergic toxicity, infection, and multiple drug overdose. It was important to explore the numerous causes of her symptoms and not just anchor on the drug overdose as the only cause of her symptoms. Workup should include blood cultures to look for bloodstream infection, urinalysis to look for urinary tract infection, chest X-ray to look for pneumonia, laboratory evaluation of alcohol level, co-ingestions, thyroid hormone, and respiratory pathogens, skin examination to check for cellulitis or abscesses, HIV testing, and echocardiogram to evaluate for vegetations from infective endocarditis given her IV drug use history.

The current case describes a rare presentation of MPP complicated by opiate overdose, requiring intubation, mechanical ventilation, targeted antibiotics, IV fluid and pressor support. This case highlights the importance of thorough physical examination and workup of undifferentiated complaints with a broad differential diagnosis in mind.

## Conclusions

Patients with MPP and respiratory failure should be monitored closely, with priority given to airway, breathing, and circulation. Targeted antibiotics therapy is the first-line treatment, with consideration of second-line agents in macrolide-resistant cases.
